# Cation Exchange in Lead Halide Perovskite Quantum Dots toward Functional Optoelectronic Applications

**DOI:** 10.1002/smsc.202300132

**Published:** 2023-11-27

**Authors:** Chenyu Zhao, Junwei Shi, Hehe Huang, Qian Zhao, Xuliang Zhang, Jianyu Yuan

**Affiliations:** ^1^ Institute of Functional Nano & Soft Materials (FUNSOM) Soochow University 199 Ren-Ai Road, Suzhou Industrial Park Suzhou 215123 P. R. China; ^2^ Department of Materials Science and Engineering Southern University of Science and Technology Shenzhen 518055 P. R. China; ^3^ School of Materials Science and Engineering Nankai University Tianjin 300350 P. R. China; ^4^ Jiangsu Key Laboratory for Carbon-Based Functional Materials & Devices Soochow University Suzhou Jiangsu 215123 P. R. China; ^5^ Jiangsu Key Laboratory of Advanced Negative Carbon Technologies Soochow University Suzhou Jiangsu 215123 P. R. China

**Keywords:** cation exchanges, optoelectronic applications, perovskite quantum dots, solar cells, stabilities

## Abstract

Lead halide perovskite quantum dots (PQDs) exhibit properties tunability and solution processability, rending them highly promising for optoelectronic applications. To overcome the compositional limits of thin‐film perovskite and achieve mixed A‐site PQDs, a post‐synthetic cation‐exchange process, driven by the intrinsic ionic character as well as the dynamic surface structure within the PQDs, emerges as a highly efficient approach. The cation‐exchange process can be precisely regulated by manipulating PQD‐situated environment, such as the cation species, stoichiometric ratios, and surface ligand conditions, leading to tunable optical bandgap, improved stability, and enhanced carrier lifetime over the single A‐site PQDs. These advancements hold immense potential for elevating the performance of PQD‐based optoelectronic devices. In this perspective, a timely summary and outlook on the emergence and developments of cation exchange in functional PQDs is presented, as well as the intrinsic cation‐exchange mechanism and properties of these resultant‐mixed‐cation PQDs. It is believed that these detailed discussions are beneficial for advancing further development of cation exchange and utilization of mixed‐cation PQDs toward functional optoelectronic applications.

## Graphical abstract

Post cation exchange, driven by intrinsic ionic properties and surface dynamics, allows efficient synthesis of mixed A‐site perovskite quantum dots (PQDs), and such a process can further optimize their optoelectronic properties and related device performance. This perspective summarizes the recent progress, delves into mechanisms, and explores mixed‐cation PQDs applications, offering insights for future development.

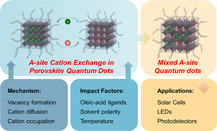

## 1 Introduction

Solution‐processable lead halide perovskite has attracted tremendous attentions in both scientific and industrial field. Meanwhile, the emergence of thin‐film perovskite semiconductors has brought about an immense interest in their corresponding perovskite quantum dots (PQDs). Such new kinds of solution‐processable PQDs have also gain increasing interests in recent years due to its remarkable properties, including high defect tolerance, near‐unity photoluminescence quantum yield (PLQY), and narrow emission linewidth.^[^ ^1^, ^2^, ^3^, ^4^, ^5^ ^]^ In addition, they exhibit tunable band gaps and an ability to stabilize the otherwise unstable crystal phases in thin‐film perovskite. In specific, the emission peak of PQDs is easily tunable across the full visible light spectrum by simply changing their chemical compositions, and PQDs are stable in the perovskite phase and can be stored for months in ambient conditions due to its high surface area and surface strain.^[^ ^1^ ^]^ These unique properties have fueled extensive research endeavors to explore their potential in the next‐generation optoelectronic applications, such as solar cells, light‐emitting diodes (LEDs) and photodetectors.^[^ ^6^, ^7^, ^8^, ^9^ ^]^

Quite recently, the upsurge of mixed A‐site thin‐films perovskite with elevated thermal and phase stability has brought about an intense interest in their counterparts of mixed‐cation PQDs.^[^ ^10^ ^]^ In general, mixed‐cation thin‐film perovskite was prepared by directly blending A‐site cation precursor. However, due to different crystallization temperatures for the pure‐component, mixed‐cation thin‐film perovskites in arbitrary compositions have remained significantly challenging.^[^ ^3^ ^]^ In contrast, colloidal PQDs with dynamic surface ligands, high surface‐to‐volume ratio, and “soft” lattice provide a good platform for designing mixed‐cation perovskites through ion exchanges. Under the circumstances, a feasible post‐synthetic cation exchange in PQDs was developed to fulfill a compositional tuning of mixed‐cation PQDs, which even overcomes the phase segregation that the corresponding perovskite thin films undergo.^[^ ^11^, ^12^ ^]^ In comparison with conventional chalcogenide and pnictide nanocrystals, the emerging ternary PQDs offer a highly accessible platform for ion exchange due to their ionic nature.^[^ ^13^, ^14^ ^]^ The rapid movement of ions within the perovskite lattice, coupled with the fast exchange dynamics in solution, enables efficient ion‐exchange reactions.^[^ ^15^, ^16^ ^]^ However, the mechanism of cation exchange still remains in the resultant observations rather than the thermodynamics analysis. To date, the cation vacancy‐assisted exchange model^[^ ^17^, ^18^ ^]^ is recognized by the perovskite community to understand the exchange process. Quite complicatedly, according to recent reports, the post‐synthetic cation‐exchange reaction is affected by lots of factors, such as system temperature, solvent polarity, ligand concentration of the PQD solution, etc. Therefore, utilizing the appropriate characterization technique to track the exchange process is necessary for in‐depth understanding of the exchange kinetics and mechanism. Benefitting from the unique defect tolerance of perovskite, precise modulation of this process involving the generation and filling of cation vacancies not only does not negatively affect the resultant‐mixed‐cation PQDs, but also can passivate surface defects, thus improving the stability and optoelectronic properties relative to single A‐site cation PQDs.

To date, the mixed‐cation colloidal PQDs have garnered significant attention as potential photoactive materials, especially in the application of the PQD solar cells (PQDSC). As mentioned earlier, A‐site mixed PQDs can effectively stabilize the crystal structure by adjusting the ratio of A‐site cations such as Cs^+^/formamidinium (FA^+^) to tailor the value of Goldschmidt tolerance factors (GTF).^[^ ^11^ ^]^ Additionally, mixed‐cation PQDs including organic compositions like FA^+^ or methylammonium (MA^+^) display longer carrier lifetime due to the fast rotation of organic cation results in enhanced orbital overlap.^[^ ^19^, ^20^ ^]^ Therefore, rational design the compositions of mixed‐cation PQDs can thoroughly enhance the performance of the mixed‐cation PQD devices in terms of carrier transport and operational stability. In this perspective, we will highlight the unique potential of mixed‐cation PQDs through cation‐exchange process, from intrinsic mechanism, recent progress to targeted optoelectronic application. We will discuss the current state of the art, various deciding factors in cation‐exchange process, challenges, and opportunities in mixed‐cation PQD materials research, and the related present and future pursuits in mixed‐cation PQDs preparation and functional optoelectronic applications.

## 2 The Progress of Cation Exchange in PQDs

Great success has been witnessed in the mixed‐cation thin‐film perovskite solar cells with the best power conversion efficiency (PCE) now exceeding 26%,^[^ ^21^, ^22^, ^23^, ^24^, ^25^, ^26^, ^27^ ^]^ promoting the rise of research on cation exchange in their counterparts of PQDs. In comparison with halide exchange, its analogous A‐site cation exchange in PQDs remains relatively unexplored so far. In this section, we will summary the progress of A‐site cation exchange in PQDs from four aspects: the emerging/synthesis of mixed A‐site PQDs, cation‐exchange kinetics, characterization of mixed‐cation PQDs, and influencing factors in the cation‐exchange process.

### 2.1 The Emergence of Cation Exchange in PQDs

**Figure 1:**
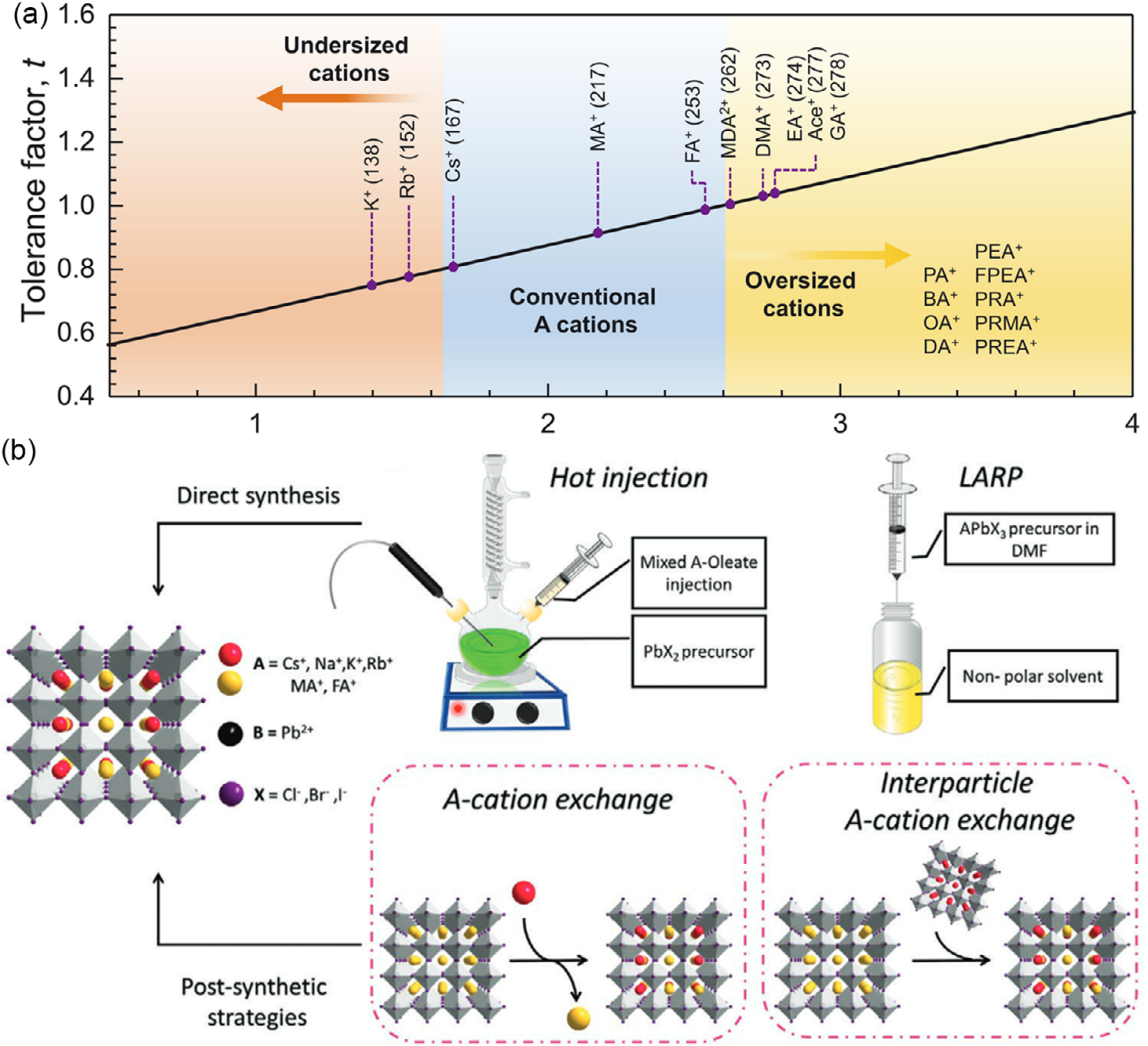
a) Mapping of tolerance factors for different ion sizes. Reproduced with permission.^[^ ^30^ ^]^ Copyright 2022, American Association for the Advancement of Science. b) Different synthesis methods diagram of mixed A‐site perovskite quantum dots (PQDs). Reproduced with permission.^[^ ^28^ ^]^ Copyright 2022, Wiley‐VCH.

Mixed‐cation PQDs are commonly synthesized using the hot injection method or the ligand‐assisted reprecipitation method (LARP), wherein the cation precursors are directly mixed for the subsequent synthesis.^[^ ^28^ ^]^ A‐site cation additives such as Na^+^, K^+^, and Rb^+^ ions are often introduced into the precursor solution to form mixed‐cation PQDs, as shown in **Figure** **1**a.^[^ ^29^, ^30^ ^]^ However, due to their small size, these ions typically occupy interstitial positions within the lattice and cannot solely replace the common Cs^+^, MA^+^, and FA^+^ ions to form the perovskite structure based on the GTF. Similarly, larger ions like Guanidine ions (GA^+^) are unable to penetrate into the interior of PQD lattices to act as A‐site cations, so they are commonly employed as surface passivation ligands or as spacer cations in 2D perovskite.^[^ ^30^ ^]^ In contrast, direct synthesis of mixed‐cation PQDs using Cs^+^, MA^+^, and FA^+^ ions is still limited due to significant differences in the formation energies among the three materials, resulting in component loss and severe phase separation. To address these challenges, LARP has been employed with utilizing antisolvents to enable crystallization of common cation sources at room temperature as illustrated in Figure 1b.^[^ ^31^ ^]^ Chen et al. successfully achieved mixed Cs_1−*m*_FA_*m*_PbBr_3_ (0 < *m* < 1) PQDs by mixing the CsBr and FABr as precursors using this approach.^[^ ^32^ ^]^ Meanwhile, the research demonstrated that incorporating PQDs with mixed Cs/FA cations exhibited improved structural stability and higher PLQY than single‐cation PQDs. Building upon these findings, this method was further extended to incorporate various cations, resulting in the successful synthesis of Cs_1−*m*_MA_*m*_PbBr_3_, Cs_1−*m*_FA_*m*_PbBr_3_, and FA_1−*m*_MA_*m*_PbBr_3_ PQDs.^[^ ^32^, ^33^ ^]^ However, due to the different crystallization conditions of perovskites with different A‐site cations, challenges still remain in accurately controlling the composition and the size distribution of the resulting PQDs.

In this regard, the post‐synthetic cation‐exchange process has been proved to be an effective approach for obtaining mixed‐cation PQDs with adjustable composition and excellent size distribution. The cation‐exchange process involves the introduction of an additional cation source into a preexisting single‐cation PQDs solution, utilizing concentration gradients and controlled chemical environments to achieve the exchange process. Two primary methods have been employed for cation exchange as shown in Figure 1b. The first method entails the utilization of cation salt solution. For instance, Protesescu et al. added Cs–oleate (Cs–OA) and FA–OA as cation sources that were added to CsPbI_3_ or FAPbI_3_ PQDs for obtaining mixed A‐site Cs_1−*m*_FA_*m*_PbI_3_ PQDs.^[^ ^3^ ^]^ The resulting PQDs exhibit bandgap values intermediate between those of their pure CsPbI_3_ or FAPbI_3_ PQDs counterparts. However, accurately quantifying and regulating the cation composition in this method is still a challenge due to the limitations in precisely controlling the preparation of Cs–OA and FA–OA. The second method involves the direct mixing of two different single‐cation PQDs. Luther et al. pioneered this approach in 2018, achieving the synthesis of mixed‐cation Cs_1−*m*_FA_*m*_PbI_3_ PQDs by blending the purified CsPbI_3_ with FAPbI_3_ PQDs.^[^ ^11^ ^]^ This method allows continuous tuning of the absorption onset and photoluminescence (PL) emission peak positions of the resulting mixed‐cation PQDs, spanning the spectral range of approximately 650–800 nm. These cation‐exchange strategies present the valuable avenues to precisely control the compositions and optical properties of mixed‐cation PQDs. Further refinement and exploration of these techniques hold significant potential for expanding the repertoire of attainable compositions and advancing the performance of PQD‐based optoelectronic devices.

### 2.2 Kinetic Process of the Cation‐Exchange Reaction

**Figure 2:**
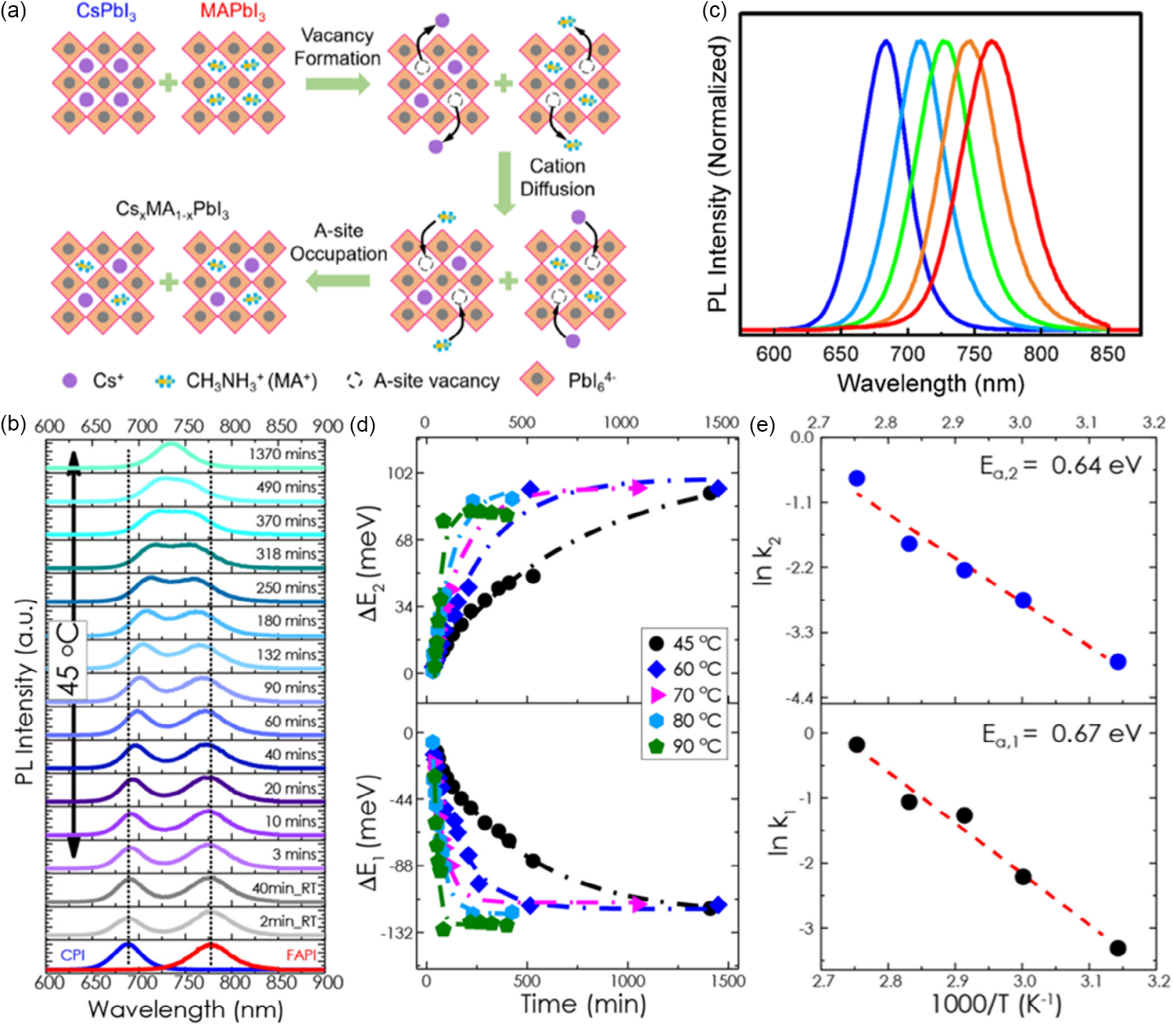
a) Schematic of vacancy‐assisted cation‐exchange process.^[^ ^34^ ^]^ Reproduced with permission. Copyright 2023, Wiley‐VCH. b) Photoluminescence (PL) evolution of CsPbI_3_ and FAPbI_3_ PQDs with time under 45 °C. c) PL spectra tuning of Cs_*m*_FA_1−*m*_PbI_3_ PQDs. d) Temperature‐depended PL emission energy shift under 45–90 °C. e) Kinetic extraction of the exchange process of Arrhenius plot for the activation energy. Reproduced with permission.^[^ ^11^ ^]^ Copyright 2018, American Chemical Society.

Although increasing research has been focused on cation exchange to obtain the mixed‐cation PQDs with improved thermal and phase stability, the mechanism of the exchange dynamics has not been comprehensively elucidated. Currently, the cation vacancy‐assisted exchange model is considered as a reliable framework for understanding this process.^[^ ^34^ ^]^ To illustrate this model, the exchange between CsPbI_3_ and MAPbI_3_ PQDs is taken as an example, as displayed in **Figure** **2**a. Initially, the ionic nature of the perovskite materials in solution causes the cation to migrate toward the periphery of the lattice, resulting in the occurrence of A‐site vacancies. Subsequently, when the different PQDs come into contact, Cs^+^ cations diffuse toward the MAPbI_3_ along a concentration gradient, occupying the vacancies previously held by MA^+^ cations. Similarly, MA^+^ cations occupy the Cs^+^ vacancies. In addition, the exchange between external ions and the parent compound involves ion migration driven by entropy.^[^ ^35^ ^]^ The aforementioned two exchange processes occur simultaneously, reaching an equilibrium state and ultimately yielding a homogeneous composition of Cs_1−*m*_MA_*m*_PbI_3_ PQDs with continuous changes in PL spectra.

To track the cation‐exchange process of PQDs, various characterization techniques are carried out. Due to high vacuum requirements and potential structure damage by the high energy electron beam, however, the cation‐exchange process in PQDs cannot be directly observed by electron microscopy.^[^ ^36^ ^]^ However, this process can be monitored by observing changes in the position of the PL emission peak.^[^ ^11^, ^37^ ^]^ Upon mixing the two different PQDs, two distinct peaks corresponding to CsPbI_3_ (680 nm) and FAPbI_3_ (780 nm) are observed as shown in Figure 2c. As the aforementioned A‐site cations are gradually incorporated into the lattice, the PL peak associated with CsPbI_3_ gradually shifts to a longer wavelength due to the introduction of FA^+^ cations, while the peak corresponding to FAPbI_3_ shifts toward shorter wavelength. Unlike the anion‐exchange process that normally completed within a few minutes, the two peaks in cation exchange exhibit minimal shift from their initial positions (associated with CsPbI_3_ and FAPbI_3_) at room temperature for a duration of approximately 40 min. This relatively slow rate of cation exchange is attributed to the larger size of the cations, which impedes their mobility. Consequently, more than 20 h are required for the two peaks to merge into one single peak at room temperature, resulting in the formation of Cs_1−*m*_FA_*m*_PbI_3_ PQDs (shown in Figure 2c). Sometimes, the peaks remain sufficiently close to each other, but the exchange process is not yet complete. In some cases, monitoring the exchange process may require analysis of the full width at half maximum of the PL emission spectra, which is commonly used in anion‐exchange studies.^[^ ^16^ ^]^

Activation energy is an important parameter to quantitatively evaluate the ion‐exchange barrier, which can be extracted from the evolution of the time‐ and temperature‐depended PL emission peaks.^[^ ^15^, ^16^ ^]^ Luther et al. systematically investigated the dynamics of the exchange between CsPbI_3_ and FAPbI_3_ from 45 to 90 °C.^[^ ^11^ ^]^ As shown in Figure 2d,e, the cation‐exchange process that initially took 20 h at 45 °C can be completed in only 2 h at 90 °C, indicating a pronounced temperature dependence of the reaction rate during cation exchange. To quantify the changes in emission energy (or bandgap) with time, which is indicative of the exchange process, the observed peak shifts can be calibrated and fitted with a single exponential function of time for all temperatures. This fitting procedure allowed the extraction of the rate at which the emission energies shifted (denoted as *k*). Fitting with the Arrhenius equation, $k=A_{0}\text{exp}\left[-\frac{E_{a}}{k_{B}T}\right]$, where $k_{B}$ is the Boltzmann constant, *T* is the absolute temperature, and $A_{0}$is the pre‐exponential factor, the activation energy $E_{a}$ (≈0.65 eV) associated with the cation‐exchange process between CsPbI_3_ and FAPbI_3_ PQDs could be quantitatively determined, which is relatively higher than that of anion exchange (≈0.45 eV)^[^ ^15^ ^]^ in PQDs.

### 2.3 Characterization of the Mixed‐Cation PQDs Prepared by Cation Exchange

**Figure 3:**
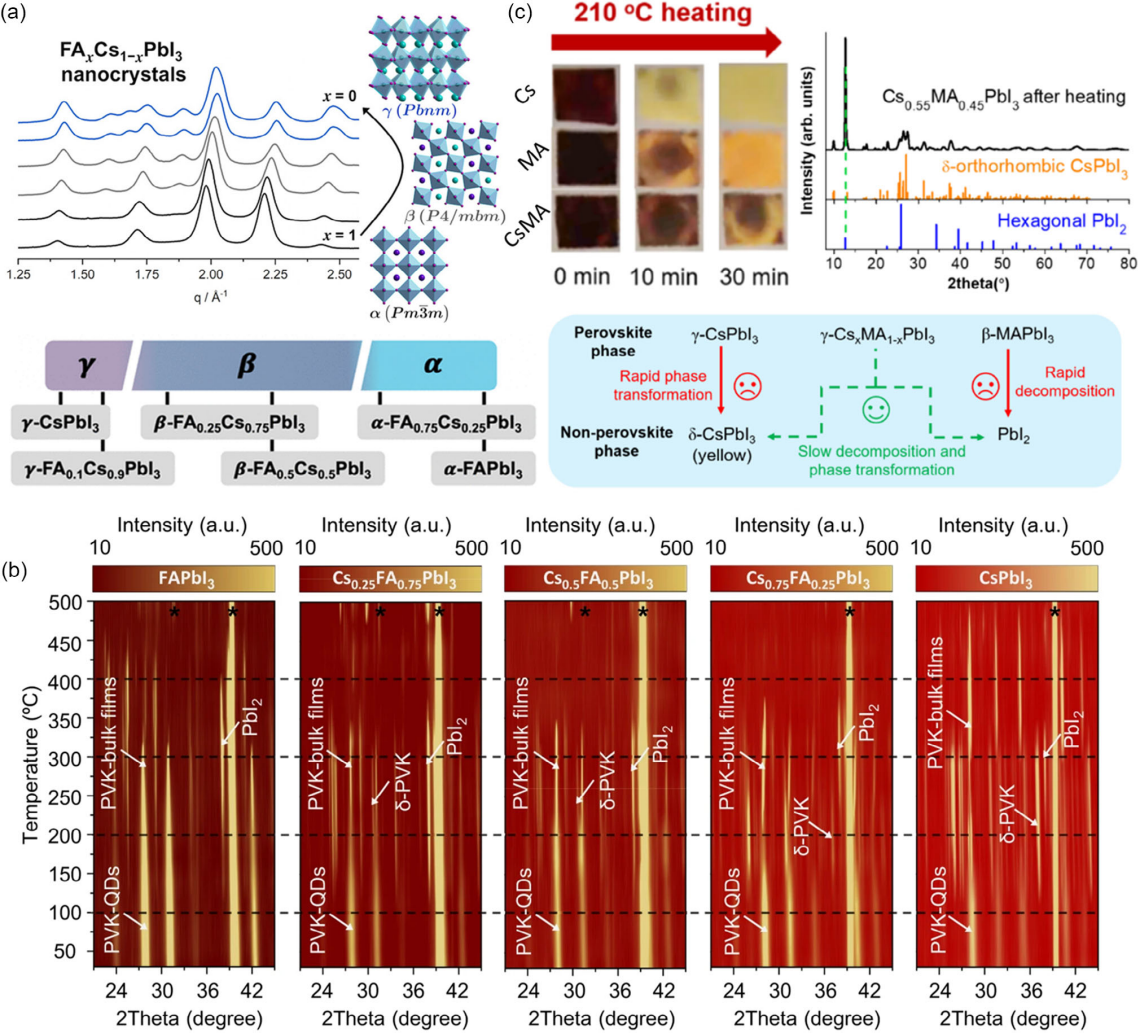
a) Crystal structure of different composition of FA_*m*_Cs_1−*m*_PbI_3_ PQD. Reproduced with permission.^[^ ^39^ ^]^ Copyright 2020, American Chemical Society. b) In situ temperature‐changing XRD analyses of FA_*m*_Cs_1−*m*_PbI_3_ PQDs. Reproduced with permission.^[^ ^40^ ^]^ Copyright 2023, Springer Nature. c) Crystal structure transformation under heating conditions (150 °C) of CsPbI_3_, MAPbI_3_, and Cs_*m*_MA_1−*m*_PbI_3_ PQDs. Reproduced with permission.^[^ ^34^ ^]^ Copyright 2023, Wily‐VCH.

In addition to utilizing the PL peak to characterize the composition of mixed‐cation PQDs, time‐resolved PL spectroscopy offers insight into the mixing of different A‐site cations within the PQD lattice. It has been observed that the excited state decay of FA‐based PQDs is significantly slower than pure Cs‐based PQDs. Therefore, the PL decay time of Cs/FA‐mixed‐cation PQDs falls between CsPbI_3_ and FAPbI_3_.^[^ ^12^ ^]^ Similarly, due to differences in lattice constants, the X‐ray diffraction (XRD) peaks of mixed‐cation PQDs are concentrated between the respective peaks of their individual counterparts, and the XRD patterns and PL emission spectra of all compositions vary linearly with the desired compositions according to the Vegard's law.^[^ ^11^, ^12^ ^]^ In a study by Vigil et al., they carefully investigated the crystal‐phase changes of FAPbI_3_ PQDs after A‐site cation exchange with Cs^+^ was carefully investigated.^[^ ^38^, ^39^ ^]^ The introduction of new A‐site cations into the lattice leads to lattice shrinkage or expansion, depending on the size of the cations, and thus affects the inclination of the [PbI_6_]^4−^ octahedra within the crystal structure. **Figure** **3**a shows that the structural symmetry of Cs_1−*m*_FA_*m*_PbI_3_ PQDs transitions from the α phase (cubic phase, *m* = 1−0.75) to *β* phase (tetragonal phase, *m* = 0.75−0.25) and γ phase (quadrature phase, *m* = 0.25–0) with increasing Cs content. Moreover, Wang et al. provided a detailed investigation of the thermal degradation process of Cs_1−*m*_FA_*m*_PbI_3_ PQDs using serial in situ probing techniques as shown in Figure 3b.^[^ ^40^ ^]^ They found that the Cs‐rich condition is dominated by a phase transition from black γ‐phase (≈30–130 °C) to yellow δ‐phase (≈130–300 °C) and then finally to black α‐phase (≈300–450 °C), while FA‐rich PQDs are mainly decomposed to PbI_2_, showing similar or even slightly higher thermal stability than Cs‐rich PQDs. To better unearth the effects of cation exchange on the structural symmetry of PQDs, the mixed‐cation PQD composed of other cations should be further studied. As shown in Figure 3c, Zhang et al. investigate the structural transformation dynamics of Cs_1−*m*_MA_*m*_PbI_3_ PQDs with γ‐phase perovskite crystal structure.^[^ ^34^ ^]^ For the cases of single A‐site cation parent PQDs, MAPbI_3_ PQDs will decompose into PbI_2_ under sustained thermal stress at 75–90 °C due to the high volatility of MA^+^, while CsPbI_3_ undergoes a transformation from γ phase to a yellow δ non‐perovskite phase at 190–200 °C. However, for Cs_0.55_MA_0.45_PbI_3_ PQDs, the aforementioned phase transition occurs at a slower rate compared to their individual counterparts, demonstrating the improved stability of mixed‐cation PQDs.

**Figure 4:**
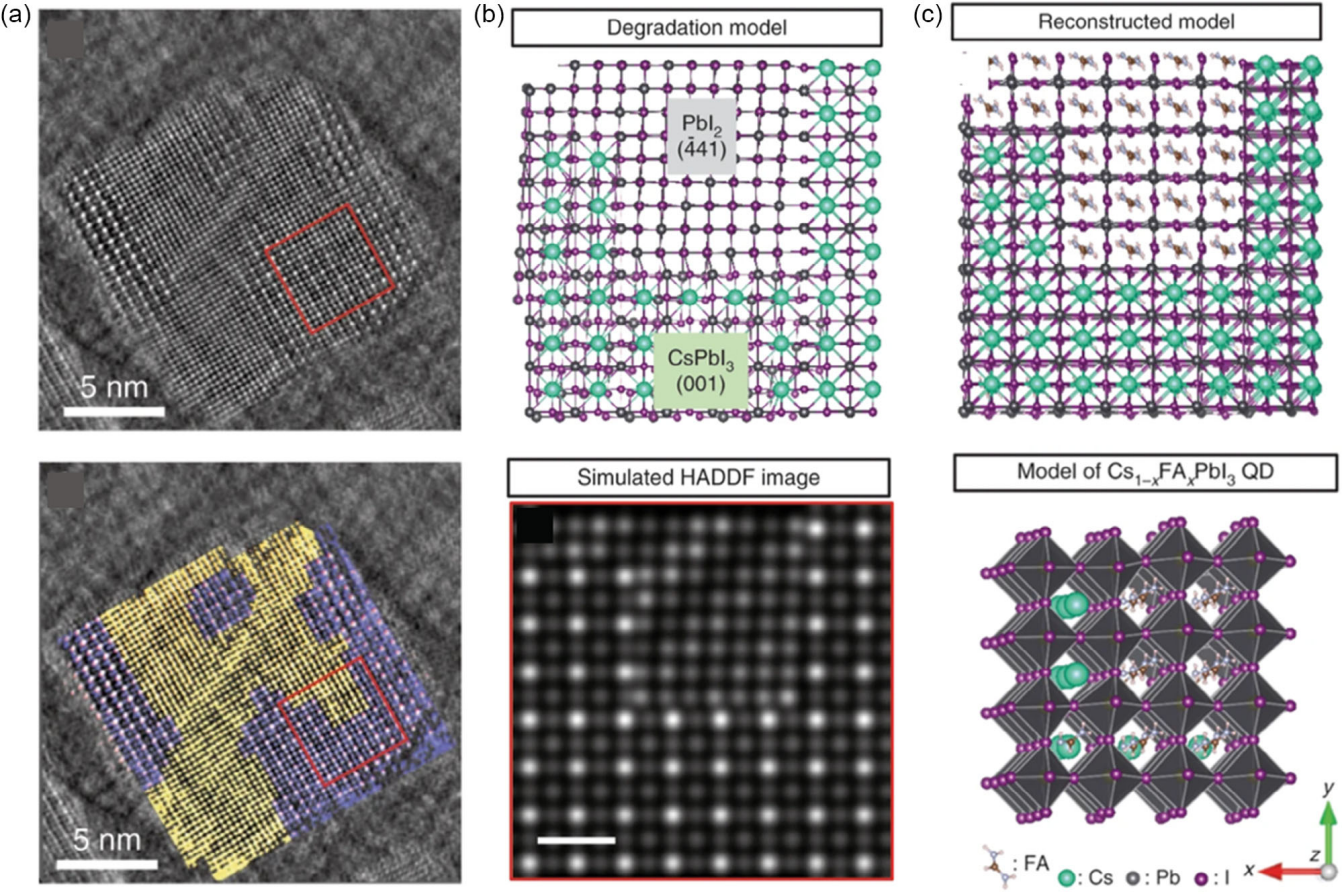
a) Original high‐angle annular dark‐field scanning transmission electron microscopy (HAADF‐STEM) images of Cs_0.5_FA_0.5_PbI_3_ PQDs. b) Simulated model of atom distribution and simulated HAADF image. c) Reconstructed atomic model of HAADF‐STEM images. Reproduced with permission.^[^ ^12^ ^]^ Copyright 2020, Springer Nature.

High‐angle annular dark‐field scanning transmission electron microscopy (HAADF‐STEM) is a commonly employed technique to study the size distribution and lattice arrangement of PQDs.^[^ ^41^, ^42^ ^]^ Hao et al. selected mixed‐cation Cs_0.5_FA_0.5_PbI_3_ PQDs and applied the HAADF‐STEM to resolve the variations in the original crystal structure of the mixed‐cation PQDs and the distribution of various cations within the lattice.^[^ ^12^ ^]^ The observed arrangement of atomic columns, as depicted in **Figure** **4**a,b, can be interpreted as a combination of CsPbI_3_ along the [001] direction and PbI_2_ along the [441] direction, with an approximate area ratio of 1:1, consistent with the exchange ratio of CsPbI_3_ and FAPbI_3_. By reconstructing an atomic model, the region corresponding to PbI_2_ with uniform atomic contrast distribution along the [441] direction can be attributed to the original FAPbI_3_ along the [001] direction because the high‐energy electron beam destroyed the FAPbI_3_ crystal structure shown in Figure 4c. Hence, it is reasonable to assume that FA and Cs cations participating in the cation‐exchange reaction are distributed within the [PbI_6_]^4−^ octahedra at shared angles, resulting in the assembly of smaller FAPbI_3_ and CsPbI_3_ units, and these units are randomly assembled in 3D space to form cubic‐shaped mixed‐cation Cs_1−*m*_FA_*m*_PbI_3_ PQDs. Quite recently, Shen and coworkers developed a low‐dose electron imaging model by applying integrated differential‐phase‐contrast STEM to atomically resolve the structure of PQDs, which avoids damage to the structure by high‐energy electron beam.^[^ ^42^ ^]^

Additionally, Li et al. conducted a study on the hot carrier cooling kinetics of pure FAPbI_3_, MAPbI_3_, CsPbI_3_, and mixed‐cation FA_0.5_MA_0.5_PbI_3_, FA_0.5_Cs_0.5_PbI_3_, and MA_0.5_Cs_0.5_PbI_3_ PQDs using ultrafast transient absorption spectroscopy.^[^ ^43^ ^]^ Coupled with the temperature‐dependent PL spectra, the intensity of electron–photon coupling was extracted. The resultants indicate that the initial fast cooling phase (<1 ps) lifetime of all organic cation PQDs is shorter than that of CsPbI_3_ PQDs. The prolonged lifetime of the slow cooling phase in the mixed‐cation PQDs, under more than 1 sun illumination, can be attributed to the induction of the covibration optical phonon mode within the mixed‐cation PQDs. These findings presents a novel strategy for slowing hot carrier cooling and provide preliminary insights into the material design of future perovskite‐based PQDs and optoelectronic technologies.

### 2.4 The Influence Factors in Cation‐Exchange Process

#### 2.4.1 The Role of Surface Ligands

**Figure 5:**
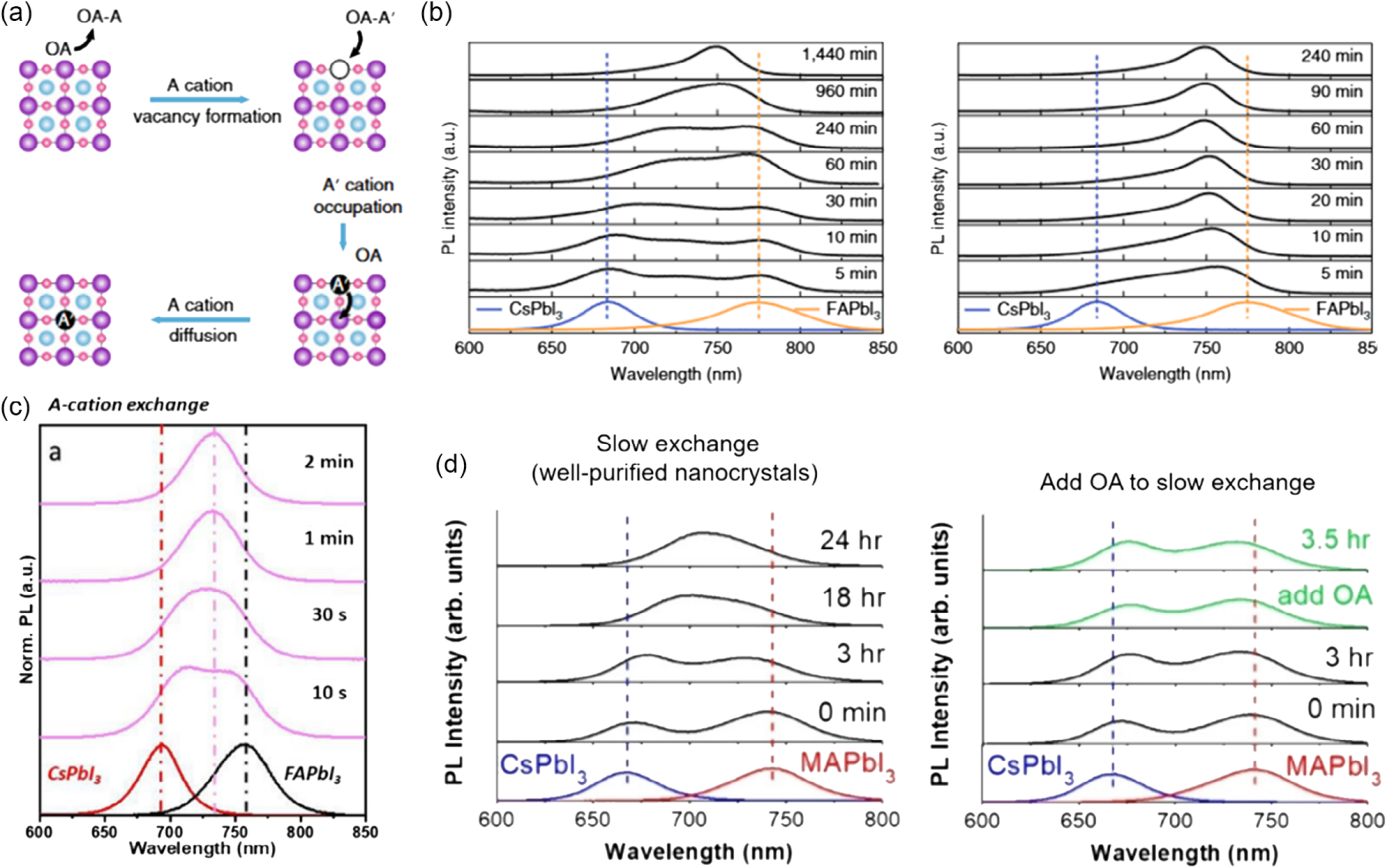
a) Diagram of OA^−^‐assisted cation‐exchange process. b) PL evolution of CsPbI_3_ and FAPbI_3_ under different surface ligand condition (left is poor OA^−^ condition, right is rich OA^−^ condition). Reproduced with permission.^[^ ^12^ ^]^ Copyright 2020, Springer Nature. c) PL evolution of cation exchange between CsPbI_3_ and FAPbI_3_ PQDs using pristine PQDs with excessive surface ligands. Reproduced with permission.^[^ ^44^ ^]^ Copyright 2022, Wiley‐VCH. d) PL evolution of cation exchange between CsPbI_3_ and MAPbI_3_ PQDs under poor ligands condition and OA‐addition condition. Reproduced with permission.^[^ ^34^ ^]^ Copyright 2023, Wiley‐VCH.

Cation exchange carried out at room temperature for a prolonged period is not favorable to the process, as it may hinder the attainment of homogeneously mixed‐cation PQDs. Meanwhile, elevating the exchange temperature to speed up the process will adversely affect the quality of the PQDs due to their temperature instability. To achieve a fast cation‐exchange process without damaging the PQDs, Hao et al. propose an oleic acid (OA) ligand–assisted cation‐exchange strategy to synthesize Cs_1−*m*_FA_*m*_PbI_3_ PQDs in a shorter time, as illustrated in **Figure** **5**a.^[^ ^12^ ^]^ Typically, single‐A‐site PQDs synthesized via the hot‐injection method cap a substantial amount of OA^−^ ligands on their surfaces. To facilitate subsequent applications, a two‐step purification process is required to remove OA^−^ ligands. Consequently, the content of OA^−^ ligands on the surface of PQDs is significantly different after one purification (rich OA^−^ ligands) and two purifications (poor OA^−^ ligands). Compared to the cation‐exchange process under poor OA‐ligand condition with over 20 h, the exchange duration for PQDs with rich OA^−^ ligands is significantly reduced to about 1 h (Figure 5b). The reduction in exchange time can be attributed to the protonation of A‐site cations (Cs^+^, FA^+^) in PQDs by OA, leading to the formation of highly mobile Cs–OA and FA–OA complexes, which eliminate the energy barriers for exchange. Furthermore, they found that the presence of abundant OA^−^ ligands during the exchange process can minimize the severe corner‐shared [PbI_6_]^4−^ octahedral distortion and preserve the lattice structure of the mixed‐cation PQDs. As a result, mixed‐cation Cs_1−*m*_FA_*m*_PbI_3_ PQDs exhibit significantly higher PL intensity and PLQY compared to those obtained under poor OA^−^ ligand conditions.

Based on the observation that OA^−^ ligands accelerate cation exchange, Otero‐Martinez et al. conducted a comprehensive investigation into the role of OA^−^ ligands in cation exchange and introduced MA^+^ cations to facilitate ternary cation exchange.^[^ ^44^ ^]^ The researchers directly mixed solutions of the synthesized single A‐site cation PQDs without any purification, resulting in the formation of mixed‐cation Cs_1−*m*_FA_*m*_PbBr_3_ PQDs. This approach involved a higher concentration of excess OA^−^ ligands, even OA monomer, in the PQDs solution. Remarkably, an ultrafast exchange process of approximately 2 min between CsPbI_3_ and FAPbI_3_ is observed in Figure 5c, comparable to that of anion exchange. Conversely, complete removal of the parent OA^−^ligands by ligand exchange with short‐chain ligands actually increases the energy barrier, thereby hindering the cation‐exchange process over 24 h. This suggests that the dynamic surface of long‐chain ligands can effectively lower the exchange energy barrier compared to short‐chain ligands. Using this method, the researchers successfully prepared mixed ternary cation Cs_1−*m*−*n*_MA_*m*_FA_*n*_PbI_3_ (0 < *m* + *n* < 1) PQDs. Additionally, Zhang et al. further investigated the addition of extra OA or OLA during the exchange of CsPbI_3_ and MAPbI_3_ and found that the mere addition of OA or OLA did not accelerate the exchange process (Figure 5d).^[^ ^34^ ^]^ Moreover, the introduction of additional OA caused a strong PL quenching of the PQDs probably due to its stronger acidity. On the contrary, the exchange between less purified PQDs with deprotonated OA^−^ exhibited an accelerated process, further confirming the effective role of OA^−^ ligand rather than OA monomer in cation exchange.

#### 2.4.2 Solvent Polarity and Other Factors

It is important to note that the cation‐exchange processes described by Luther and Otero‐Martinez exhibit non‐negligible difference in exchange durations under poor OA‐ligand condition, with the former requiring 20 h, while the latter only 1 h.^[^ ^11^, ^44^ ^]^ This notable distinction may be attributed to the variance in the polarity of the host solvent environment (n‐octane for the former and toluene for the latter) during the exchange. Although both n‐octane and toluene are considered to be common nonpolar solvents for dispersing PQDs, toluene has a slightly higher polarity than n‐octane, potentially reducing the energy barrier and accelerating the exchange process. The effect of different solvents on exchange is more commonly observed in anion exchange. For instance, Zhou et al. employed a polar adsorption strategy to achieve precise control in anion exchange.^[^ ^17^ ^]^ Solvents with higher polarity can interact more strongly with surface ions, resulting in adsorption of surface ions and acceleration of the exchange process. In another study by Zhao et al., they investigated the influence of different solvents on the exchange of well‐purified CsPbI_3_ and FAPbI_3_ PQDs.^[^ ^45^ ^]^ They also found that the use of toluene as the PQD dispersing solvent significantly accelerated the exchange compared to n‐octane, and further acceleration was observed when chloroform, a solvent with higher polarity, was employed. This observation further confirmed that the rate of the cation‐exchange process is dependent on the polarity of the solvents. However, it is important to note that increasing the polarity of the solvent will severely reduce the phase stability of PQDs, especially CsPbI_3_ PQDs. When chloroform or more polar solvents were used, CsPbI_3_ rapidly agglomerated and transformed into an undesired non‐perovskite phase. Therefore, it is crucial to harmonize a suitable exchange condition that allows a fast process while ensuring high quality mixed‐cation PQDs.

In addition to the influence of solvent and ligands, the introduction of A‐site salt solution during PQD purification will also affect the exchange process. Recently, Jia et al. reported a feasible antisolvent‐assisted in situ cation‐exchange method for precise modulation of Cs/FA composition in the PQDs.^[^ ^46^ ^]^ In this approach, they used a solution of acetonitrile/toluene with formamidinium iodide (FAI) salts to substitute methyl acetate for the purification of PQDs, in which the high polarity of acetonitrile enables the dissolution of substantial amounts of FAI salts, which serve as a sufficient FA^+^ cation source for the exchange. As mentioned earlier, meanwhile, the synergy of the high polarity of acetonitrile and excess OA^−^ ligands facilitates rapid exchange within minutes, while acetonitrile also aids in the removal of the OA/OLA ligands from the PQDs surface without compromising their quality. Additionally, the excess iodide ions present in FAI can compensate for iodine deficiencies resulting from the purification process. Consequently, this approach achieves a significantly reduced exchange duration and enables the production of Cs_1−*m*_FA_*m*_PbI_3_ PQDs with adjustable Cs/FA composition. However, it should be noted that excessively high concentrations of FAI in the acetonitrile/toluene solution can cause severe agglomeration of PQDs, thereby impeding comprehensive regulation of continuous Cs/FA composition. Nevertheless, this method is an effective approach to promote cation exchange in PQDs.

## 3 Cation Exchange in PQDs for Functional Optoelectronic Applications

Long‐chain surface ligands and defects severely impede charge carrier transport between dot to dot, while the mixed‐cation PQDs incorporating organic components exhibit enhanced carrier lifetime and reduced non‐radiative recombination. Beyond that, the mixed‐cation PQDs show relatively higher thermal and structural stability compared to their corresponding mono‐cation PQDs. Therefore, the mixed‐cation PQDs are potential candidates for functional optoelectronic applications, including solar cells, LEDs, detectors, etc.

### 3.1 PQD Solar Cells

To date, the emerging colloidal PQDs, especially the mixed‐cation PQDs, have garnered significant attention as the potential photoactive materials for the next‐generation solar cells due to their high light absorption coefficient and defect tolerance, as well as improved phase stability.^[^ ^47^, ^48^ ^]^ Among them, all‐inorganic CsPbI_3_ PQDs have been extensively investigated owing to their quantum size‐induced surface strain, which leads to the stabilization of the black phase of CsPbI_3_ perovskite at low temperatures. Significant progress has been made in enhancing the photovoltaic performance of inorganic PQDSC, with the PCE increasing rapidly from an initial 10.7% to over 16% in recent years, achieved through precise control of the surface ligand chemistry and synthesis strategies of CsPbI_3_ PQDs.^[^ ^5^, ^49^, ^50^ ^]^ However, the relatively narrow light absorption spectra (<700 nm) and inherent soft crystal structures of inorganic PQDs impose limitations on further enhancing the photovoltaic performance and operational stability of PQDSC. Therefore, reducing the bandgap (*E*_g_) and increasing the GTF of PQDs are the critical strategies to further enhance the performance of PQDSC.^[^ ^51^ ^]^ However, the existing organic FAPbI_3_ PQDs with more favorable GTF, suitable bandgap, and improved crystal stability merely achieve an efficiency of over 14%, which is still lower than that of CsPbI_3_ PQDSC.^[^ ^20^ ^]^ Moreover, both CsPbI_3_ and FAPbI_3_ PQDs exhibit high sensitivity to moisture and polar solvents during the post‐synthesis purification and surface‐treatment processes. This inherent susceptibility presents a formidable challenge in achieving effective ligand engineering and results in a significant increase in surface defects, thereby impeding carrier transport and ultimately limiting the enhancement of PCE. Consequently, in comparison to pure CsPbI_3_ and FAPbI_3_ PQDs, mixed A‐site cation Cs_1−*m*_FA_*m*_PbI_3_ PQDs offer enhanced stability and charge transport properties, which is attributed to the A‐site cation exchange that enhances the entropic stabilization of the perovskite structure even under ambient conditions.^[^ ^51^ ^]^ Furthermore, the rapid rotation of FA molecules within the lattice facilitates enhanced orbital overlap and promotes the formation of polarons, leading to prolonged carrier lifetimes and mitigated non‐radiative recombination processes.^[^ ^52^, ^53^ ^]^

**Figure 6:**
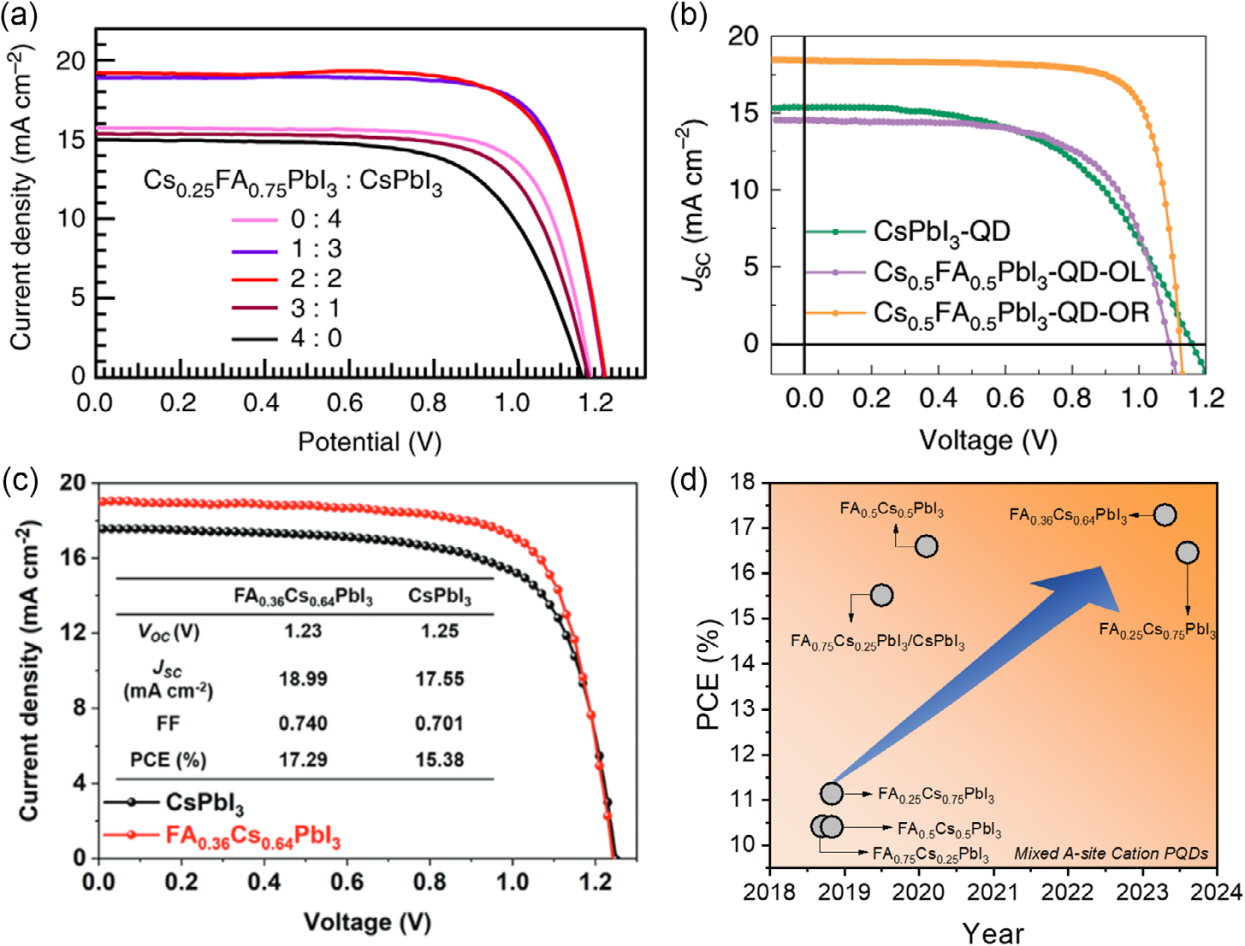
a) Current density–voltage (*J*–*V*) curves of different ratio of Cs_0.25_FA_0.75_PbI_3_ to CsPbI_3_ layer in PQDSC. Reproduced with permission.^[^ ^54^ ^]^ Copyright 2019, Springer Nature. b) *J*–*V* curves of PQDSC using different PQDs: CsPbI_3_, Cs_0.5_FA_0.5_PbI_3_–QD–PQD‐OA (less) (OL), Cs_0.5_FA_0.5_PbI_3_–QD–PQD‐OA (rich) (OR). Reproduced with permission.^[^ ^12^ ^]^ Copyright 2020, Springer Nature. c) *J*–*V* curves of Cs_0.36_FA_0.64_PbI_3_ and CsPbI_3_ PQDSC. Reproduced with permission.^[^ ^46^ ^]^ Copyright 2023, Wiley‐VCH. d) Power conversion efficiency growth of mixed A‐site PQDSC in recent years.

The as‐prepared mixed‐cation PQDs with finely tuned Cs/FA composition can be applied to optoelectronic applications such as solar cells, LEDs, and photodetectors. **Table** **1** shows the development of the power efficiency of mixed‐cation PQDSC. In 2018, Luther et al. first introduced the mixed‐cation Cs_1−*m*_FA_*m*_PbI_3_ PQDs into solar cells, which exhibit enhanced efficiencies and lower hysteresis compared to pure CsPbI_3_ PQDSC, demonstrating a PCE of mixed‐cation PQDSC of approximately 12%.^[^ ^11^ ^]^ In 2019, Zhao et. al. synthesized the mixed‐cation Cs_1−*m*_FA_*m*_PbI_3_ PQDs by directly mixing CsPbI_3_ and FAPbI_3_ PQDs and demonstrated a heterojunction solar cell with abrupt compositional changes throughout the perovskite film.^[^ ^54^ ^]^ Benefitting from a built‐in electric field, the heterojunction effectively promotes charge separation at the internal interface, thereby improving photocarrier collection and enhancing light harvesting. Eventually, the device with a 1:3 layers ratio (Cs_0.25_FA_0.75_PbI_3_: CsPbI_3_) achieved a stabilized power output (SPO) PCE of over 15.52% seen in **Figure** **6**a. In 2020, Hao et al. reported a certified PCE of 16.6% using the Cs_1−*m*_FA_*m*_PbI_3_ PQDs prepared by OA‐assisted cation exchange.^[^ ^12^ ^]^ They found that the PQDSC exhibited higher PCE with extended spectral absorption compared to the CsPbI_3_ PQDSC (as shown in Figure 6b). More importantly, the mixed Cs_0.5_FA_0.5_PbI_3_ PQDSC showed suppressed phase segregation and long‐term stability for device operation under continuous illumination of 1 sun. In 2023, Jia et al. reported an antisolvent‐assisted in situ cation‐exchange method for the preparation of Cs_1−*m*_FA_*m*_PbI_3_ PQDs, and the device yielded a champion PCE of up to 17.29%, which is the highest value among the reported homo‐structured PQDSC (as shown in Figure 6c).^[^ ^46^ ^]^ Meanwhile, due to the strong structural resistance of PQDs under ambient and continuous illumination conditions, the storage and operational stability of PQDSC are greatly enhanced. They analyzed the charge carrier dynamics of mixed‐cation PQDs films and solar cells and demonstrated that the enhanced photovoltaic performance is attributed to a combination of broadened light‐harvesting spectra, flattened energy landscape, and favorable energy levels of highly oriented PQDs film. Figure 6d shows the PCE growth trend of mixed PQDSC in recent years, a risen from 10% to 17% can be observed, which indicating huge potential of mixed A‐site cation PQDs for further application.

**Table 1:** Literature overview of device performance of mixed‐cation PQD solar cells

Composition	Year	Device structure	*V*_OC_ [V]	*J*_SC_ [mA cm^−2^]	FF	PCE [%]	References
FA_0.25_Cs_0.75_Pbl_3_	2018	FTO/TiO_2_/PQDs/Spiro‐OMeTAD/MoO_*x*_/Al	1.15	14.36	0.68	11.14	[11]
FA_0.5_Cs_0.5_Pbl_3_	2018	FTO/TiO_2_/PQDs/Spiro‐OMeTAD/MoO_*x*_/Al	1.13	14.80	0.62	10.42	[11]
FA_0.75_Cs_0.25_Pbl_3_	2018	FTO/TiO_2_/PQDs/Spiro‐OMeTAD/MoO_*x*_/Al	1.10	14.37	0.66	10.41	[11]
Cs_0.25_FA_0.75_PbI_3_/CsPbI_3_	2019	ITO/TiO_2_/PQDs/spiro‐OMeTAD/MoO_*x*_/Al	1.20	18.91	0.76	15.52 (SPO)	[54]
FA_0.5_Cs_0.5_Pbl_3_	2020	ITO/SnO_2_/PQDs/Spiro‐OMeTAD/Au	1.17	18.30	0.78	16.6	[12]
FA_0.36_Cs_0.64_PbI_3_	2023	ITO/SnO_2_/PQDs/Spiro‐OMeTAD/Ag	1.23	18.99	0.74	17.29	[46]
FA_0.25_Cs_0.75_PbI_3_/FA_0.5_Cs_0.5_PbI_3_/FA_0.75_Cs_0.25_PbI_3_	2023	ITO/SnO_2_/PQDs/Spiro‐OMeTAD/Au	1.15	19.31	0.75	16.68	[62]

In addition, Li et al. reported a methylammonium iodide (MAI)‐assisted solution‐mediated ligand exchange (SMLE) method to adjust the surface and internal chemical environment of FAPbI_3_ PQDs in solution to form mixed‐cation PQDs partially doped with MA cations.^[^ ^55^ ^]^ The MAI–SMLE treatment has a negligible effect on the crystal structure of FAPbI_3_ PQDs but can effectively reduce the density of surface ligands and passivate the trap states. Based on the aforementioned, the SMLE FAPbI_3_ PQDSC obtained a champion PCE of 15.10%, which resulted from the significant improvement in the short‐circuit current density (*J*_SC_) and fill factor (FF), which is much higher than that of the control device (12.12%). In contrast, few studies have focused on the integration of mixed‐cation PQDs into bulk thin‐film photovoltaics to improve their stability. For instance, Chen and co‐workers demonstrated that the deposition of Cs‐rich mixed‐cation Cs_1−*m*_FA_*m*_PbI_3_ PQD layer on bulk FAPbI_3_ layer improves the device stability under ambient conditions.^[^ ^56^ ^]^ The device shows negligible hysteresis compared to the device without the PQDs layer. The aforementioned resultants elucidate that the development of mixed‐cation PQDs is an effective strategy to simultaneously enhance the device performance and improve the stability of perovskite solar cells.

### 3.2 PQD LEDs and Others

**Figure 7:**
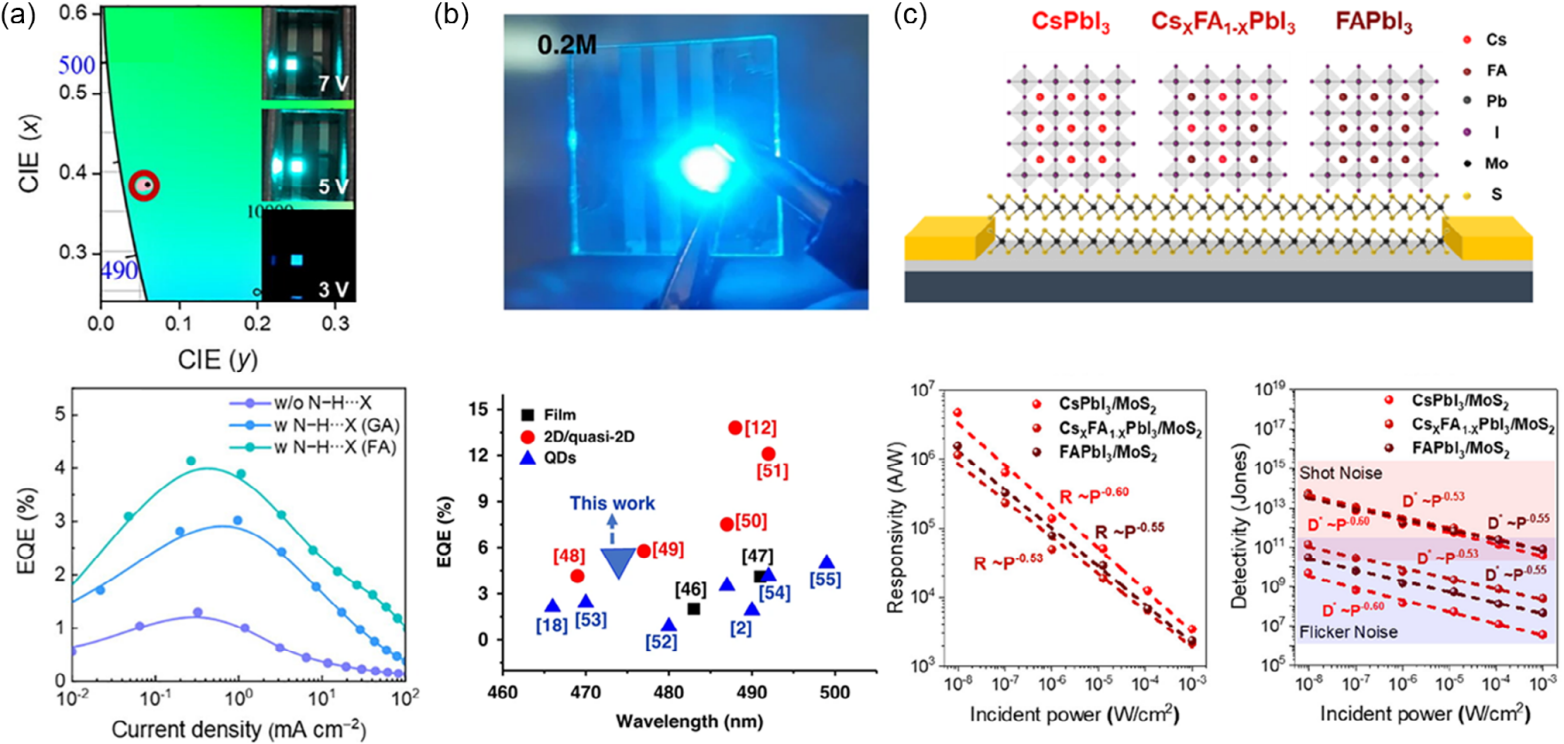
a) Commission internationale de l’eclairage (CIE) color corresponding to formamidinium (FA)‐based light‐emitting diode (LED) devices; EQE–current density data of the devices. Reproduced with permission.^[^ ^57^ ^]^ Copyright 2021, Elsevier. b) Photograph of FA‐doped LED device; EQE–wavelength data of the devices. Reproduced with permission.^[^ ^58^ ^]^ Copyright 2022, Springer Nature. c) Schematic diagram of mixed PQDs/MoS_2_ structure; responsivity and detectivity of PQDs‐based photodetectors. Reproduced with permission.^[^ ^60^ ^]^ Copyright 2023, American Chemical Society.

And beyond the solar cells, mixed‐cation PQDs are widely used in the application of LEDs for the next‐generation light‐emitting devices (commonly referred to as QLEDs). Achieving high efficiency and stable pure blue colloidal PQD LEDs is still an enormous challenge because the Cl–Br‐mixed perovskite emitters typically exhibit high defect density, low PLQY, and phase segregation.^[^ ^57^, ^58^, ^59^ ^]^ Zhang et. al achieved color stable blue PQD LEDs with improved external quantum efficiency (EQE) from 1.3% (CsPbBr_*n*_Cl_3−*n*_, 0 < *n* < 3) to 3.02% (GA_*m*_Cs_1−*m*_PbBr_*n*_Cl_3−*n*_) and 4.14% (FA_*m*_Cs_1−*m*_PbBr_*n*_Cl_3−*n*_) by a hydrogen‐bonded amine group doping strategy using guanidinium (GA^+^)‐ or FA^+^‐doping source for CsPbBr_*n*_Cl_3−*n*_ PQDs.^[^ ^57^ ^]^ The improved EQE is attributed to the strong bonding between the amine group and halide, which can be generated between −NH_2_ dopants and Pb−X lattices, thereby increasing the migration barrier of halide anions. Resultantly, as illustrated in **Figure** **7**a, color‐stable sky‐blue devices were realized, exhibiting emission peaks fixed at 490.5 nm (GA) and 492.5 nm (FA) without any obvious shift with increasing voltage, in sharp contrast to devices without N–H⋯X producing a 15 nm redshift from 487 to 502 nm. Additionally, Gao et al. reported a strategy for modifying the organic cation composition to synthesize pure blue‐emitting PQDs at room temperature.^[^ ^58^ ^]^ The synthesized FA–CsPb(Cl_0.5_Br_0.5_)_3_ PQDs exhibited bright PL with high PLQY (65%), which is six times higher than the undoped samples. In addition, the photophysical properties of FA cation doping was deeply illustrated through carrier dynamics characteristics and first principal calculations, and the doped PQDs showed the lower defect density, longer PL lifetime, and more reasonable bandgap structure than the undoped emitters. Consequently, pure blue FA–CsPb(Cl_0.5_Br_0.5_)_3_ PQDs light‐emitting devices were fabricated and presented a maximum luminance of 1452 cd m^−2^, and an EQE of 5.01% with an emission peak at 474 nm (see Figure 7b). The excellent device performance is mainly attributed to the well‐passivated PQDs emitter and effective charge injection and exciton emission.

Moreover, mixed‐cation PQDs can be used as near‐infrared light detectors. Jeong et al. reported a high‐performance 0D–2D hybrid photodetectors integrated with different bandgap PQDs (CsPbI_3_, Cs_1−*m*_FA_*m*_PbI_3_, and FAPbI_3_) and molybdenum (IV) sulfide (MoS_2_) bilayer (Figure 7c).^[^ ^60^ ^]^ In this structure, the PQDs with different compositions can be used as the absorbing layer of light with a specific wavelength and transfer the photogenerated carriers to the MoS_2_ layer. Ultimately, the photodetector with PQD layer achieved a high responsivity up to 10^7^ A W^−1^ and a high specific detectivity exceeding 10^13^ Jones.

## 4 Conclusion and Outlook

For the first time, we provide insights into the emergence and progress of cation exchange in PQDs, including the underlying exchange mechanisms, properties, deciding factors, and applications of the resultant‐mixed‐cation PQDs. The A‐site composition of colloidal PQDs can be precisely controlled by post‐synthetic cation exchange using appropriate A‐site cation precursors or cross‐exchange between PQDs with different A‐site cations, in which the exchange process can be tuned by modulating the ligand concentration and the polarity of the dispersed solvent in the PQD solution. Meanwhile, antisolvent‐assisted cation exchange, simultaneously modulating the aforementioned two factors, achieves a rapid exchange process without affecting the structural stability of the PQDs. Significantly, the as‐prepared mixed PQDs display higher phase stability and better optical properties than single A‐site cation PQDs. Therefore, solar cells based on mixed‐cation PQDs exhibit long‐term stability and higher PCE than those of single‐cation PQDSC. Meanwhile, the mixed‐cation PQDs have been widely used in other optoelectronic devices such as LEDs and photodetectors.

Although significant progress has been made in the optoelectronic applications of mixed‐cation PQDs, several challenges remain toward future high‐performing PQD‐based devices. 1) Understanding of the cation‐exchange process: currently, the mechanism of cation exchange still stays in the observation analysis. In‐depth understanding of the thermodynamic process and unraveling the distribution and movement of cations in the crystal lattice of mixed‐cation PQDs will not only help to precisely control the cation‐exchange reactions, but also unravel the origin of phase segregation in mixed‐cation bulk perovskite. 2) Innovation in surface chemical regulation: the surface ligands, temperature, and solvent environment play a crucial role in the cation‐exchange process. A detailed study of the surface chemistry of the PQD system is essential for reducing the activation energy and accelerating exchange rate to prepare high‐quality and damage‐free mixed‐cation PQDs. 3) Optimizing the performance of mixed‐cation PQDSC: rational design in the composition and surface ligand of PQDs during cation exchange can effectively improve the structural stability, passivate trap states, and facilitate carrier transport. In addition, the development of novel device architecture involving the sequential deposition of PQDs with different bandgap can realize a homogeneous energy landscape, accelerating carrier extraction. Currently, the bulk perovskites with mixed A‐site cation have been extensively studied in the next‐generation optoelectronic device. 4) Extending the A‐site cation‐exchange methods to B‐site cations: the toxicity of Pb element has always been an obstacle to the further application of perovskite materials in optoelectronic devices, and it is a feasible method to find non‐lead elements, such as Sn, Mn, etc., to replace Pb element. At present, Pb has been successfully partial replaced by Mn cation using cation‐exchange method.^[^ ^61^ ^]^ Through addressing the aforementioned challenges, we believe that the development of A‐site mixed‐cation PQDs will simultaneously enhance the material properties and performance of related optoelectronic devices.

## Conflict of Interest

The authors declare no conflict of interest.
